# Serum Fucosylated Haptoglobin as a Novel Diagnostic Biomarker for Predicting Hepatocyte Ballooning and Nonalcoholic Steatohepatitis

**DOI:** 10.1371/journal.pone.0066328

**Published:** 2013-06-21

**Authors:** Yoshihiro Kamada, Maaya Akita, Yuri Takeda, Shin Yamada, Hideki Fujii, Yoshiyuki Sawai, Yoshinori Doi, Hitomi Asazawa, Kotarosumitomo Nakayama, Kayo Mizutani, Hironobu Fujii, Takayuki Yakushijin, Masanori Miyazaki, Hisao Ezaki, Naoki Hiramatsu, Yuichi Yoshida, Shinichi Kiso, Yasuharu Imai, Norifumi Kawada, Tetsuo Takehara, Eiji Miyoshi

**Affiliations:** 1 Department of Molecular Biochemistry and Clinical Investigation, Osaka University, Graduate School of Medicine, Suita, Osaka, Japan; 2 Department of Gastroenterology and Hepatology, Osaka University, Graduate School of Medicine, Suita, Osaka, Japan; 3 aMs New Otani Clinic, Osaka, Japan; 4 Department of Hepatology, Osaka City University Graduate School of Medicine, Osaka, Japan; 5 Department of Gastroenterology, Ikeda Municipal Hospital, Ikeda, Osaka, Japan; 6 Department of Gastroenterology, Otemae Hospital, Osaka, Japan; Institute of Medical Research A Lanari-IDIM, University of Buenos Aires-National Council of Scientific and Technological Research (CONICET), Argentina

## Abstract

Nonalcoholic fatty liver disease (NAFLD) is a growing medical problem around the world. NAFLD patients with nonalcoholic steatohepatitis (NASH) can develop cirrhosis and hepatocellular carcinoma. The ability to distinguish NASH from simple steatosis would be of great clinical significance. Ballooning hepatocytes are characteristic of typical pathological NASH; here, the polarized secretion of proteins is disrupted due to destruction of the cytoskeleton. We previously reported that fucosylated glycoproteins are secreted into bile, but not into sera in normal liver. Therefore, we hypothesized that the fucosylation-based sorting machinery would be disrupted in ballooning hepatocytes, and serum fucosylated glycoproteins would increase in NASH patients. To confirm our hypothesis, we evaluated serum fucosylated haptoglobin (Fuc-Hpt) levels in biopsy-proven NAFLD patients (n = 126) using a lectin-antibody ELISA kit. Fuc-Hpt levels were significantly increased in NASH patients compared with non-NASH (NAFLD patients without NASH) patients. Interestingly, Fuc-Hpt levels showed a significant stepwise increase with increasing hepatocyte ballooning scores. Multiple logistic regression analysis showed that Fuc-Hpt levels were independent and significant determinants of the presence of ballooning hepatocytes. Moreover, Fuc-Hpt levels were useful in monitoring liver fibrosis staging. Next, to investigate the significance of serum Fuc-Hpt in a larger population, we measured Fuc-Hpt levels in ultrasound-diagnosed NAFLD subjects (n = 870) who received a medical health checkup. To evaluate NAFLD disease severity, we used the FIB-4 index (based on age, serum AST and ALT levels, and platelet counts). Fuc-Hpt levels increased stepwise with increasing FIB-4 index.

**Conclusion:**

Measurement of serum Fuc-Hpt levels can distinguish NASH from non-NASH patients, and predict the presence of ballooning hepatocytes in NAFLD patients with sufficient accuracy. These results support the potential usefulness of measuring Fuc-Hpt levels in clinical practice.

## Introduction

Nonalcoholic fatty liver disease (NAFLD) is among the most common causes of chronic liver disease in the world and is a growing medical problem in industrialized countries [1]. A wide spectrum of histological changes has been observed in NAFLD, ranging from simple steatosis (which is generally non-progressive) to nonalcoholic steatohepatitis (NASH), and a proportion of patients with NASH develop cirrhosis and hepatocellular carcinoma (HCC) [2]. Approximately 30% of the general population has NAFLD and up to 5% of the population has NASH [3]–[5]. Liver biopsy remains the gold standard for diagnosing NASH and grading the severity of liver damage [6], [7]. However, invasive liver biopsy is poorly suited as a diagnostic test for such a prevalent condition, and this in turn restricts therapeutic intervention. Moreover, biopsy itself carries significant limitations such as pain, risk of severe complications, sampling error [8], cost [9], and patient unwillingness to undergo invasive testing. Therefore, the need for development and validation of a reproducible and noninvasive test that can accurately distinguish NASH from simple steatosis is urgent.

Recent findings in glycobiology include direct evidence of the involvement of oligosaccharide changes in human diseases [10]. Glycoproteomics has been in focus as a post-genomic research field for the identification of diagnostic markers [11], [12]. In particular, fucosylation, characterized by the addition of fucose to the glycans, is an important oligosaccharide modification involved in cancer and inflammation [13]. Various fucosylated proteins are reported to be biomarkers for human diseases [14]–[16]. We previously reported that fucosylated-haptoglobin (Fuc-Hpt) is a novel marker for patients with pancreatic cancer and colon cancer [16]–[18]. Haptoglobin is an acute phase protein mainly produced in the liver, and we have previously reported that interleukin-6 (IL-6), a typical inflammatory cytokine, upregulated fucosylation regulatory genes [19]. Furthermore, we also showed that fucosylation is a possible signal for the polarized secretion of fucosylated glycoproteins into bile ducts in the liver [20]. In normal hepatocytes, Fuc-Hpt produced in the liver would be secreted into bile, and not in the sera. On the other hand, ballooning hepatocytes are known as a typical pathological characteristic of NASH and alcoholic hepatitis [21]–[23]. In ballooning hepatocytes, the microtubule cytoskeleton, which is essential for normal efficient vesicle transport in the hepatocyte, is destroyed [24]. Its destruction induces nascent protein retention and an increase in the diameter of the hepatocyte. Collectively, the fucosylation-based sorting machinery would be disrupted in the ballooning hepatocyte, and Fuc-Hpt produced in the liver would be secreted into sera. Indeed, serum Fuc-Hpt levels assessed by western blotting were elevated in patients with alcoholic liver diseases [25]. In this study, we hypothesized that serum fucosylated glycoproteins levels would be elevated in NASH patients.

In the present study, to confirm our hypothesis, we analyzed serum Fuc-Hpt levels using a lectin antibody enzyme-linked immunosorbent assay (ELISA) in biopsy-proven NAFLD patients and subjects who underwent health check-ups. Our study demonstrated that serum Fuc-Hpt levels were significantly higher in NASH patients compared with non-NASH (NAFLD patients without NASH) patients. Moreover, serum Fuc-Hpt levels were closely correlated with the presence of ballooning hepatocytes. Our findings suggest that serum Fuc-Hpt levels could be a novel and useful biomarker for noninvasive NASH diagnosis.

## Patients and Methods

### Ethics Statement

The protocol and informed consent were approved by institutional review boards at Osaka University Graduate School of Medicine. Written informed consent was obtained from all patients at the time of liver biopsy or the medical health check-up, and the study was conducted in accordance with the Helsinki Declaration.

### Biopsy-proven NAFLD Patients Study

A total of 126 patients with NAFLD confirmed by liver biopsy between 2008 and 2012 were enrolled from the following institutes: Osaka University Hospital, Ikeda Municipal Hospital, Otemae Hospital, and Osaka City University Hospital. The normal controls (NC) were sera from 24 healthy subjects who underwent a health checkup. The sera remaining after the medical checkup were used after permission had been received from the subjects. Sera from the liver biopsy-proven NAFLD patients were kept frozen at −80°C until used. The histological criterion used for the diagnosis of NAFLD was the presence of macrovesicular fatty changes in the hepatocytes, with displacement of the nuclei to the cell edge [26]. The criteria for exclusion from this study were as described below in the health checkup study.

### Chronic Hepatitis Type C Patients

To compare the serum Fuc-Hpt levels with NAFLD patients, 29 patients with chronic hepatitis type C (CHC) were enrolled. These patients were collected at Osaka University Hospital from 2009 to 2012. Sera were taken at the time of liver biopsy, and histological evaluation was performed.

### Medical Health Check-up Study

Among 1,500 Japanese adult subjects (1,131 males, 369 females) who underwent health checkups at aMs New Otani Clinic (Osaka, Japan) from 2008 to 2009, 1,044 subjects (797 male, 247 female) were recruited into this study. The criteria for exclusion from this study included a history of hepatic disease, such as chronic hepatitis C or concurrent active hepatitis B (seropositive for hepatitis B surface antigen), autoimmune hepatitis, primary biliary cirrhosis, sclerosing cholangitis, hemochromatosis, α1-antitrypsin deficiency, Wilson’s disease, or hepatic injury caused by substance abuse, as well as a current or past history of consumption of more than 20 g of alcohol daily. Among the 1,044 subjects, 870 subjects (659 male, 211 female) were diagnosed with fatty liver, and 174 subjects (138 male, 36 female) were diagnosed without fatty liver by abdominal ultrasound. Serum from the subjects were collected at the health checkup and kept frozen at −80°C until used. The FIB-4 index (based on age, serum aspartate [AST] and alanine aminotransferase [ALT] levels, and platelet counts) was calculated for each of the subjects as previously reported (age × AST (U/L)/platelet count (×10^9^/L)/√ALT (U/L)) [27], [28]. The FIB-4 index has been demonstrated to be superior to other noninvasive markers of fibrosis in Japanese NAFLD patients [29]. The cutoff values proposed by Shah et al. [28] were adopted in this study (low cutoff point, 1.30; high cutoff point, 2.67).

### Anthropometric and Laboratory Evaluation

Anthropometric variables (height and weight) were measured using a calibrated scale after requesting the patients to remove their shoes and any heavy clothing. Body mass index (BMI) was calculated as weight (kg) divided by the square of height in meters (m^2^). Venous blood samples were obtained in the morning after the patients had fasted overnight for 12 hours. Laboratory evaluations for all patients included determination of platelet counts, and measurement of the serum levels of AST, ALT, γ-glutamyl transpeptidase (GGT), albumin, total cholesterol, triglyceride, fasting blood glucose, immunoreactive insulin (IRI), ferritin, and hyaluronic acid. All of the parameters were measured using standard techniques.

### Histological Evaluation

All patients enrolled in this study had undergone percutaneous liver biopsy under ultrasound guidance. The liver specimens were embedded in paraffin and stained with hematoxylin and eosin and Masson’s trichrome stains. Two hepatic pathologists (Y.K. and H.F.) who were blinded to the clinical data reviewed the liver biopsy specimens. Adequate liver samples were defined as >1.5 cm long and/or having more than six portal tracts. NASH was defined according to Matteoni’s classification [30]. Patients whose liver biopsy specimens showed simple steatosis or steatosis with non-specific inflammation were placed in the “non-NASH” cohort. Samples were also investigated and quantified according to NAFLD activity scoring (NAS) [22]. Steatosis (0–3), lobular inflammation (0–3), and hepatocellular ballooning (0–2) were quantified. The individual parameters of fibrosis were scored independently according to the NASH Clinical Research Network scoring system [22]. Advanced fibrosis was classified as a stage 2–4 disease. In our study, decisions about each pathologic feature and the diagnosis of NASH were made by consensus between two hepatic pathologists [31]. Briefly, we diagnosed ballooning hepatocytes using HE staining as enlarged (>30 µm in diameter) with pale staining and a rarefied cytoplasma and rounded cell shape [32].

### Lectin-antibody ELISA for Fuc-Hpt

The Fab fragment of antihuman haptoglobin IgG (Dako, Carpinteria, CA) was coated onto the bottom of a 96-well ELISA plate, because IgG has a fucosylated oligosaccharide in its Fc portion. Coated plates were blocked with phosphate-buffered saline (PBS) containing 3% bovine serum albumin for 1 hour, followed by washing with PBS containing 0.1% Tween 20 (PBS-T). A 50-µL aliquot of sera was placed into each well and incubated for 1 hour at room temperature. The plate was washed three times with PBS-T, using Immuno Wash (Bio-RAD Model 1517, Hercules, CA). To detect Fuc-Hpt, 1/1000 diluted biotinylated Aleuria aurantia lectin was placed into each well, followed by incubation at room temperature for 1 hour. After washing the plates three times with PBS-T, peroxidase-conjugated avidin was added to each well, followed by incubation at room temperature for 1 hour. After washing four times with PBS-T, tetramethylbenzidine was added to each well, followed by a 15-minute incubation for development. To stop the development, 1 N sulfuric acid was added to each well. A standard curve for Fuc-Hpt was obtained as previously described [19], using a Fuc-Hpt standard purchased from Takara Bio Inc. (Shiga, Japan).

### ELISA for Haptoglobin, and Caspase-cleaved Cytokeratin-18 (M30 Antigen)

Haptoglobin was measured using the AssayMax Human Haptoglobulin ELISA kit (Assaypro, St. Charles, MO) in accordance with the manufacture’s protocol. For the quantitative measurement of the caspase-generated neoepitope of cytokeratin-18, we used the M30-Apoptosense ELISA (Peviva, Bromma, Sweden) according to the instructions of the manufacturer.

### Statistical Analysis

Statistical analysis was conducted using JMP 9.0 software (SAS Institute Inc Cary, NC). Continuous variables were expressed as mean ± standard deviation. Qualitative data were represented as numbers, with the percentages indicated in parentheses. Kruskal-Wallis tests and Wilcoxon tests were used to assess whether there were any significant differences in terms of continuous clinical or serological characteristics between groups. Chi-square tests were used for categorical factors. Spearman’s correlation coefficient was used to estimate the association of serum Fuc-Hpt and several factors of interest. The diagnostic performances of the scoring systems were assessed by analyzing the receiver operating characteristic (ROC) curves. The probabilities of a true positive (sensitivity) and true negative (specificity) assessment were determined for selected cutoff values and the area under the receiver operating characteristic curve (AUROC) was calculated for each index. The Youden index was used to identify the optimal cutoff points. Multivariate logistic regression analyses were conducted to identify parameters that significantly contribute to the estimation of hepatocyte ballooning scores. Differences were considered statistically significant at *P*<0.05.

## Results

### Study of Biopsy-proven NAFLD Patients

At first, to investigate the significance of serum Fuc-Hpt levels in NAFLD patients, we measured serum Fuc-Hpt levels in biopsy-proven NAFLD patients (n = 126). The clinical and biochemical characteristics of individuals in this study are shown in Table 1. Significant differences were found between non-NASH and NASH groups in age (*P*<0.01), AST (*P*<0.01), IRI (*P*<0.05), ferritin (*P*<0.05), platelet count (*P*<0.01), hyaluronic acid (*P*<0.01), FIB-4 index (*P*<0.01), Fuc-Hpt (*P*<0.01), and M30 antigen (*P*<0.05). There were no significant differences between the two groups with respect to gender, BMI, ALT, AST/ALT ratio, GGT, total cholesterol, triglyceride, glucose, and albumin. The levels of serum haptoglobin also were not significantly different (*P* = 0.9945). The distribution of Matteoni's classification among the 126 patients is shown in Table S1. The histological characteristics of liver biopsy specimens from NAFLD patients (non-NASH and NASH) are shown in Table S2.

**Table 1:** Clinical and serological characteristics of the biopsy-proven NAFLD patients.

Factor	All subjects (n = 126)	nonNASH (n = 19)	NASH (n = 107)	*P* Value*
**Age (yr)**	54.4±12.8	46.9±13.6	55.7±12.3	<0.01
**Gender (M/F)**	70/56	13/6	57/50	0.2207
**BMI (kg/m^2^)**	27.5±5.1	27.0±4.1	27.5±5.2	0.5646
**AST (U/L)**	62.9±39.3	44.2±36.5	66.3±39.0	<0.01
**ALT (U/L)**	95.8±72.0	71.9±48.8	100.1±74.7	0.0809
**AST/ALT ratio**	0.74±0.28	0.64±0.21	0.76±0.29	0.0752
**GGT (U/L)**	111.8±117.2	146.5±172.8	105.5±104.2	0.8392
**Total cholesterol (mg/dL)**	200.2±38.7	209.5±27.8	198.6±40.2	0.1690
**Triglyceride (mg/dL)**	152.8±78.7	145.4±60.6	154.2±81.8	0.8933
**Glucose (mg/dL)**	113.3±36.8	110.3±35.3	113.9±37.2	0.5067
**IRI (mU/mL)**	13.7±11.3	9.28±4.69	14.38±11.84	<0.05
**Albumin (g/dL)**	4.14±0.43	4.26±0.30	4.12±0.44	0.1187
**Ferritin (µg/dL)**	334.1±358.1	181.4±140.8	362.6±379.0	<0.05
**Platelet count (x10^9^/L)**	200.3±64.3	248.8±67.1	191.7±60.1	<0.01
**Hyaluronic acid (ng/dL)**	73.0±91.9	26.5±17.8	81.1±97.1	<0.01
**FIB-4 index**	2.11±1.47	1.02±0.70	2.30±1.49	<0.01
**Fuc-Hpt (U/mL)**	573.8±965.7	111.1±202.1	655.9±1023.6	<0.01
**M30 antigen (U/L)**	811.3±780.8	573.6±511.1	854.3±814.6	<0.05
**Haptoglobin (mg/dL)**	89.3±29.5	89.4±29.9	89.3±29.5	0.9945

Data are presented as the mean ± SD.

Abbreviations: BMI, body mass index; AST, aspartate aminotransferase; ALT, alanine aminotransferase; GGT, gamma glutamyl transpeptidase; IRI, immunoreactive insulin; Fuc-Hpt, fucosylated haptoglobin.

* *P* values correspond to the comparison between nonNASH and NASH group. Wilcoxon test for continuous factors and Pearson's Chi-square test for categorical factors were used.

Next, we investigated the correlation coefficients of relationships between serum Fuc-Hpt levels and various parameters in the NAFLD patients (Table S3). Serum Fuc-Hpt levels showed significant and positive correlations with AST/ALT ratio (r = 0.31, *P*<0.01), hyaluronic acid (r = 0.55, *P*<0.01), FIB-4 index (r = 0.40, *P*<0.01), hepatocyte ballooning scores (r = 0.41, *P*<0.01), and fibrosis stage (r = 0.41, *P*<0.01). Fuc-Hpt levels had significant and negative correlations with platelet count (r = −0.32, *P*<0.01) and haptoglobin (r = −0.27, *P*<0.01). Fuc-Hpt levels had significant and positive correlations with NAS (r = 0.28, *P*<0.01) and inflammation scores (r = 0.26, *P*<0.01). There was no significant correlation between Fuc-Hpt levels and steatosis scores (r = −0.16, *P* = 0.075). Serum M30 antigen levels are known to be higher in NASH patients and their use in the diagnosis of NASH has been proposed [33], [34]. There was no correlation between Fuc-Hpt and M30 antigen levels in NAFLD patients (r = 0.056, *P* = 0.54) (Table S3).

### Fuc-Hpt Levels are Significantly Increased in NASH Patients

**Figure 1:**
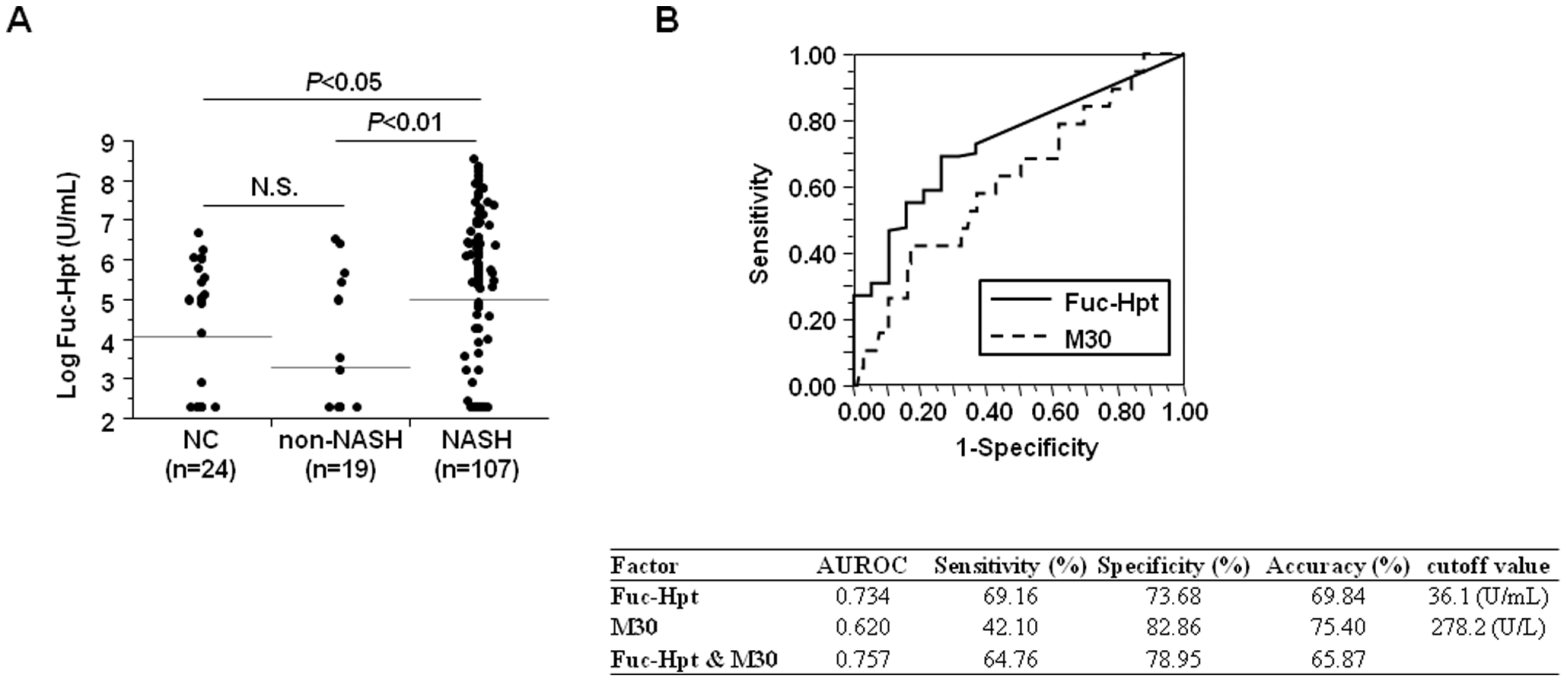
Serum Fuc-Hpt levels were significantly elevated in biopsy-proven NASH patients. (A) Serum Fuc-Hpt levels in each group (normal controls, non-NASH patients, NASH patients). Horizontal grey lines indicate the mean values of Fuc-Hpt in each group. NC, normal controls. (B) ROC curves for Fuc-Hpt and the M30 antigen for the discrimination of NASH.

We examined whether serum Fuc-Hpt levels were useful for the diagnosis of NASH. There was no significant difference in Fuc-Hpt levels between normal control and non-NASH patients (167.8±211.0 vs. 111.1±202.1 U/mL, *P* = 0.221) (Table 1, Fig. 1A). Importantly, serum Fuc-Hpt levels in NASH patients exhibited greater increases than did those in non-NASH patients (655.9±1023.6 U/mL, *P*<0.01). In our study, serum M30 antigen levels were also significantly higher in NASH patients than in non-NASH patients (Table 1).

**Figure 2:**
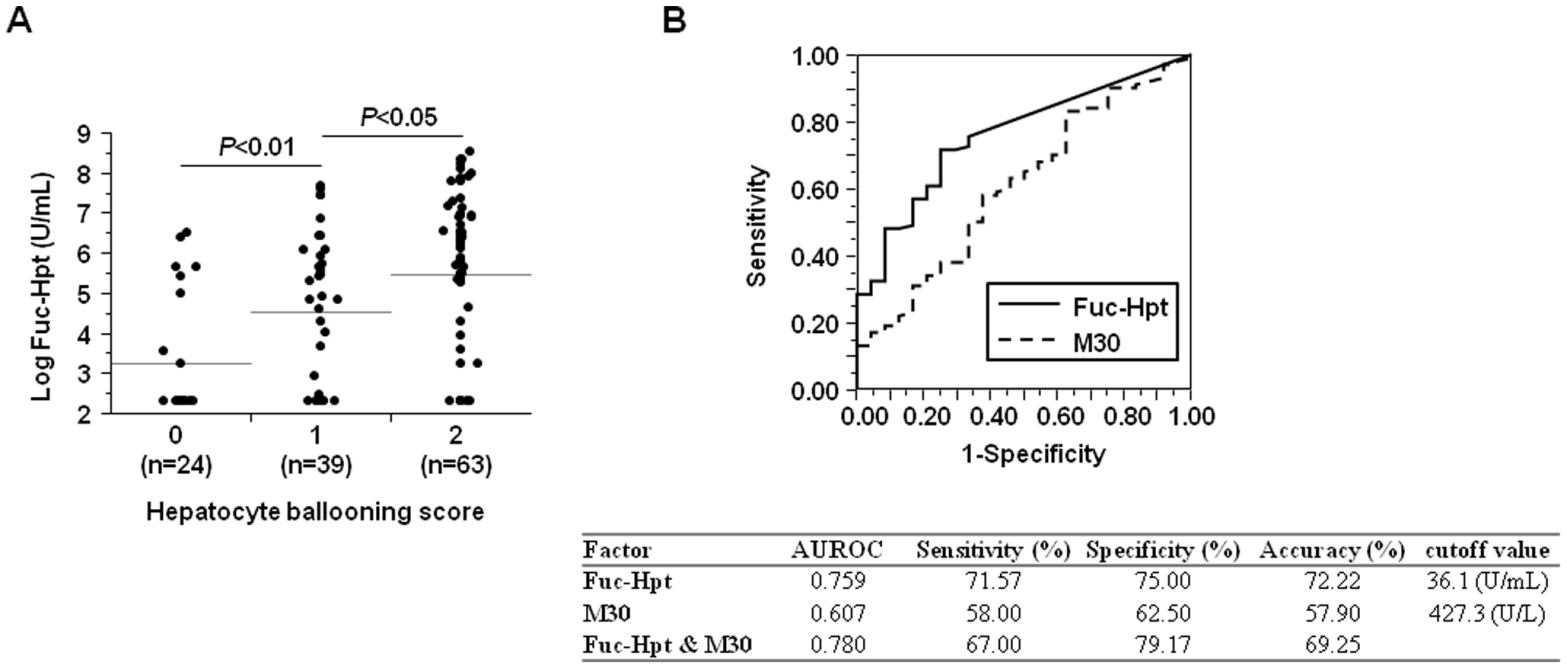
Correlation between serum Fuc-Hpt levels and hepatocyte ballooning scores in biopsy-proven NAFLD patients. (A) Serum Fuc-Hpt levels in each group classified by hepatocyte ballooning scores. (B) ROC curves for Fuc-Hpt and the M30 antigen for the presence of ballooning hepatocytes.

**Figure 3:**
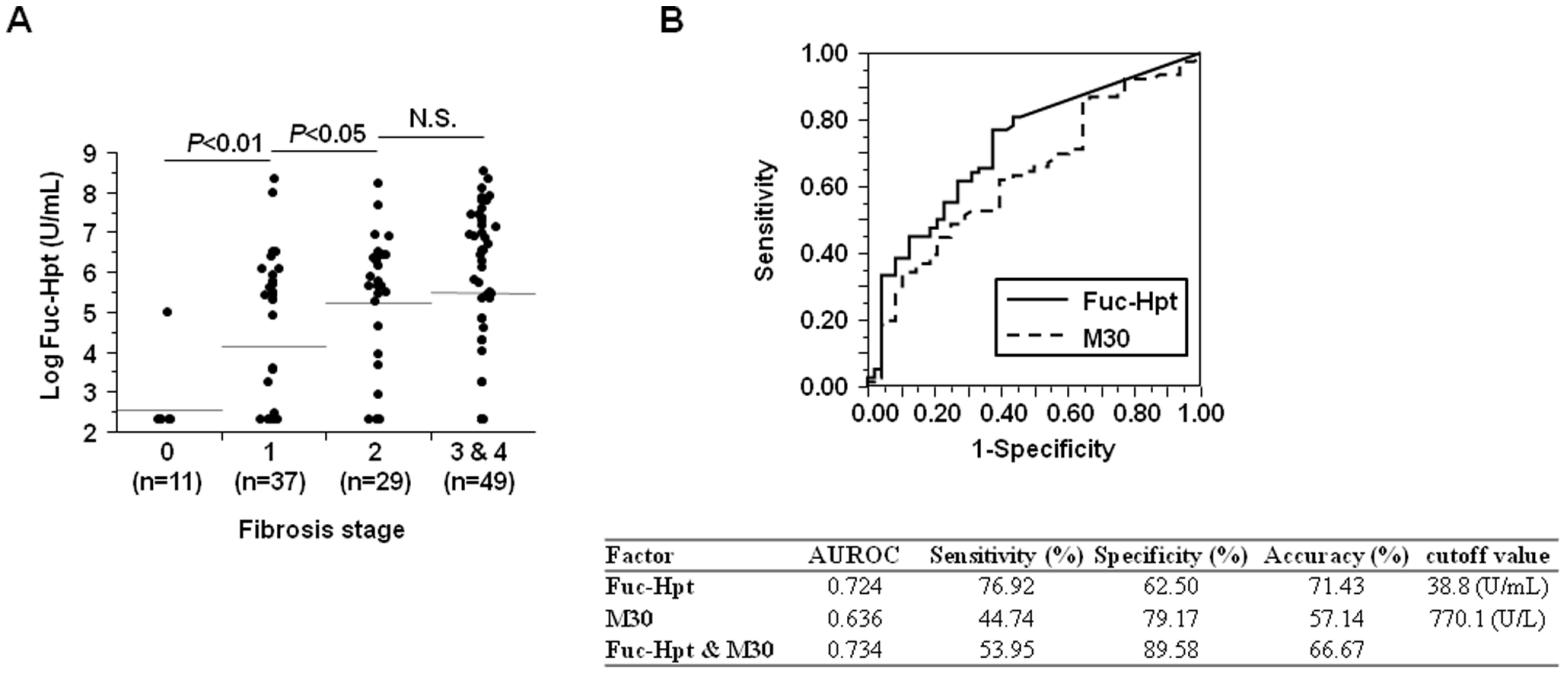
Correlation between serum Fuc-Hpt levels and liver fibrosis stage in biopsy-proven NAFLD patients. (A) Serum Fuc-Hpt levels in each stage of liver fibrosis in NAFLD patients. (B) ROC curves for Fuc-Hpt and the M30 antigen for discrimination of advanced liver fibrosis in biopsy-proven NAFLD patients.

Among NAS, Fuc-Hpt levels showed a significant and stepwise increase with increased hepatocyte ballooning scores (Table 2, Fig. 2A), and fibrosis stage progression (F0; 22.7±42.0, F1; 353.6±813.0, F2; 512.7±771.0, F3 and 4; 899.9±1169.7 U/mL, *P*<0.01) (Fig. 3A). In addition, serum Fuc-Hpt levels had significant correlations with both typical serum fibrosis markers (platelet count, hyaluronic acid, FIB-4 index) and the histological fibrosis score (Table S3).

**Table 2:** Clinical and serological characteristics of the biopsy-proven NAFLD patients classified by hepatocyte ballooning score.

	Hepatocyte ballooning score			
Variables	0	1	2	*P* value
**Number**	24	39	63	
**Age**	46.1±12.7	52.9±12.2	58.4±11.7	<0.01
**Gender (M/F)**	13/11	27/12	29/34	0.0734
**BMI**	26.9±4.0	27.4±4.2	27.7±5.9	0.8026
**AST**	43.5±32.6	66.4±42.1	68.2±38.1	<0.01
**ALT**	78.0±47.0	106.7±84.8	95.9±70.9	0.4322
**AST/ALT ratio**	0.60±0.21	0.71±0.24	0.81±0.31	<0.01
**GGT**	133.4±157.4	102.0±81.4	109.6±119.2	0.9181
**Total cholesterol**	214.6±29.5	200.1±40.0	194.9±40.1	0.0633
**Triglyceride**	153.2±62.6	157.4±89.9	149.9±78.2	0.7822
**Glucose**	107.1±32.2	112.1±41.9	116.5±35.3	<0.05
**IRI**	9.45±4.97	10.97±5.62	17.20±14.55	<0.05
**Albumin**	4.27±0.28	4.22±0.45	4.04±0.44	<0.05
**Ferritin**	233.3±267.9	301.4±286.2	392.0±46.0	<0.05
**Platelet count**	250.2±61.5	193.5±55.5	185.6±61.8	<0.01
**Hyaluronic acid**	25.1±16.1	58.8±61.4	99.9±113.5	<0.01
**FIB-4 index**	0.97±0.64	2.07±1.42	2.57±1.50	<0.01
**Fuc-Hpt**	101.5±187.0	365.1±573.6	882.9±1206.2	<0.01
**M30 antigen**	586.0±493.6	720.3±690.1	954.3±895.8	0.17
**Haptoglobin**	88.1±29.1	93.5±30.7	87.3±29.1	0.5526

Data are presented as the mean ± SD.

M30 antigen levels have been reported to correlate with NASH diagnosis, hepatocyte ballooning scores, and fibrosis severity in NAFLD patients [34]. We also compared the sensitivity and specificity of Fuc-Hpt with those of M30 antigen for the discrimination of NASH, the presence of ballooning hepatocytes (hepatocyte ballooning score 0 vs. 1 and 2), and the discrimination of advanced fibrosis (F0 & F1 vs. F2–4) using the ROC curve and the AUROC (Fig. 1B, 2B, 3B). All the AUROCs of Fuc-Hpt were higher than that of the M30 antigen for the detection of NASH, ballooning hepatocyte presence, and advanced liver fibrosis (0.734 vs. 0.620, 0.759 vs. 0.607, and 0.724 vs. 0.636, respectively). The cutoff values for Fuc-Hpt were 36.1 U/mL (NASH diagnosis), 36.1 U/mL (ballooning hepatocyte discrimination), and 38.8 U/mL (fibrosis severity prediction). The cutoff values for the M30 antigen were 278.2 U/L (NASH diagnosis), 427.3 U/L (ballooning hepatocyte discrimination), and 770.1 U/L (fibrosis severity prediction). The sensitivity of Fuc-Hpt was much higher than that of the M30 antigen for the differentiation of NASH and non-NASH (69.16 vs. 42.10%). However, the specificity of Fuc-Hpt was lower than that of the M30 antigen (73.68 vs. 82.86%). The accuracy of Fuc-Hpt was almost the same as that of the M30 antigen (69.84 vs. 75.40%) (Fig 1B). The sensitivity and specificity of Fuc-Hpt were higher than those of M30 antigen for the differentiation of the presence of hepatocyte ballooning (71.57 vs. 58.00%, 75.00 vs. 62.50%, respectively). The accuracy of Fuc-Hpt was also higher than that of M30 antigen (72.22 vs. 57.90%) (Fig. 2B). The sensitivity of Fuc-Hpt was much higher than that of M30 antigen for the differentiation of fibrosis severity (76.92 vs. 44.74%), while the specificity was lower (62.50 vs. 79.17%). The accuracy of Fuc-Hpt was higher than that of M30 antigen (71.43 vs. 57.14%) (Fig. 3B). The combination of Fuc-Hpt and M30 didn't enhance the significance of the detection of NASH, ballooning hepatocyte presence, and advanced liver fibrosis. Since these cut-off values for Fuc-Hpt were quite low, we adopted several cutoff values, and analyzed their sensitivity, specificity, and accuracy for the detection of NASH, ballooning hepatocyte presence, and advanced liver fibrosis (Table S4A, B, C).

Next, we further evaluated the significance of serum Fuc-Hpt levels on the presence of ballooning hepatocytes by multiple logistic regression analysis (Table 3). Age, BMI, AST, GGT, total cholesterol, triglyceride, IRI, ferritin, platelet count, Fuc-Hpt, and M30 antigen were selected as variables based on multivariate analysis. We found that age, AST, platelet count, and Fuc-Hpt levels were independent and significant determinants for the discrimination of the presence of ballooning hepatocytes. M30 antigen levels were not a significant determinant in our study.

**Table 3:** Multiple logistic regression analysis of factors associated with hepatocyte ballooning score 1–2 compared to score 0.

		95% CI		
Factor	Odds ratio	Lower	Upper	*P* value
**Age**	1.0737	1.0036	1.1596	<0.05
**BMI**	1.0343	0.8749	1.2624	0.7053
**AST**	1.0183	1.0001	1.0417	<0.05
**GGT**	0.9964	0.9892	1.0022	0.2326
**Total cholesterol**	0.9902	0.9670	1.0143	0.4102
**Triglyceride**	0.9991	0.9867	1.0128	0.8861
**IRI**	1.0755	0.9719	1.2688	0.1877
**ferritin**	0.9996	0.9969	1.0033	0.8133
**Platelet count**	0.9792	0.9600	0.9940	<0.01
**Fuc-Hpt (per 100U/mL increase)**	1.4196	1.0171	2.7146	<0.05
**M30**	1.0000	0.9989	1.0014	0.9386

### Comparison of Serum Fuc-Hpt Levels between NAFLD and Chronic Hepatitis Type C Patients

In order to investigate serum Fuc-Hpt levels in other liver disease, we compared serum Fuc-Hpt levels between NAFLD and CHC patients (Figure S1). The data indicated that serum Fuc-Hpt levels increased with the fibrosis stage progression in both NAFLD and CHC patients. In CHC patients, serum Fuc-Hpt levels increased especially in patients with advanced fibrosis (F3). Interestingly, NAFLD patients with hepatocyte ballooning score 2 showed high levels of serum Fuc-Hpt levels even in F1 and F2 stage patients. These results indicated that serum Fuc-Hpt levels cannot be used to distinguish NASH from CHC patients with advanced fibrosis, but could be a useful biomarker for the discrimination of NASH patients with early liver fibrosis. The presence of ballooning hepatocytes could elevate serum Fuc-Hpt levels in NASH patients.

### Medical Health Check-up Study

To investigate the significance of serum Fuc-Hpt levels in a larger population, we measured serum Fuc-Hpt levels in NAFLD subjects (n = 870) who received health checkups. Table 4 lists the basic anthropometric data and the results of biochemical tests of all NAFLD subjects enrolled in the health checkup study. These subjects had not received liver biopsy, so we evaluated the hepatic disease severity of NAFLD subjects using the FIB-4 index in the health checkup study [27], [28]. The FIB-4 index is based on age, serum AST and ALT levels, and platelet counts; these parameters are usually measured in health checkups in Japan. In addition, the FIB-4 index has been reported to be superior to other noninvasive markers of fibrosis in NAFLD patients [29]. The clinical and biochemical characteristics of individuals in the health checkup study classified by FIB-4 index categories (proposed by Shah et al. [28]) are shown in Table 5. Age (*P*<0.01), AST (*P*<0.01), AST/ALT ratio (*P*<0.01), GGT (*P*<0.01), and Fuc-Hpt (*P*<0.01) levels revealed significant stepwise elevation, while total cholesterol (*P*<0.01) and platelet count (*P*<0.01) showed significant stepwise decrease with progressively higher FIB-4 index categories. Multivariate logistic regression analysis indicated that serum Fuc-Hpt levels were independent and significant determinants for the prediction of F3 (by FIB-4 index) (odds ratio, 1.183; 95% CI 1.057–1.324; *P*<0.01) (Table S5). These results indicate that measurements of serum Fuc-Hpt levels could also predict NAFLD severity in a large population.

**Table 4:** Clinical and biochemical characteristics of NAFLD subjects in the health check-up study.

Variables	NAFLD subjects
**Number**	870
**Age (yr)**	55.3±6.8
**Gender (M/F)**	659/211
**BMI (kg/m^2^)**	26.2±3.6
**SBP (mmHg)**	120.8±15.6
**DBP (mmHg)**	73.0±10.7
**AST (U/L)**	31.6±17.6
**ALT (U/L)**	43.4±25.0
**AST/ALT ratio**	0.87±0.39
**GGT (U/L)**	64.5±71.5
**Albumin (g/dL)**	4.4±0.2
**Total cholesterol (mg/dL)**	211.0±35.0
**Triglyceride (mg/dL)**	152.0±93.2
**HDL-C (mg/dL)**	53.9±11.4
**Glucose (mg/dL)**	122.0±34.1
**Creatinine (mg/dL)**	0.83±0.36
**Uric acid (mg/dL)**	6.0±1.3
**Iron (µg/dL)**	114.7±38.8
**Platelet count (x10^9^/L)**	222.4±53.0
**FIB-4 index**	1.32±0.72
**Fuc-Hpt (U/ml)**	190.6±235.7

Data are presented as the mean ± SD.

**Table 5:** Clinical and serological characteristics of the NAFLD subjects in health check-up study classified by FIB-4 index categories.

	FIB-4 index (cutoff values proposed by Shah et al.)			
Variables	Low cutoff point (<1.30)	Indeterminate (1.30–2.67)	High cutoff point (>2.67)	*P* value
**Number**	525	315	30	
**Age**	53.2±5.7	58.2±6.9	62.2±8.7	<0.01
**Gender (M/F)**	391/134	244/71	24/6	0.5283 (chi square test)
**BMI**	26.4±3.7	25.9±3.5	25.2±4.3	0.0585
**SBP**	119.7±15.7	122.3±15.5	122±15.0	<0.05
**DBP**	73.1±11.0	73.2±10.2	70.1±10.3	0.201
**AST**	26.9±9.6	36.3±16.7	64.9±53.2	<0.01
**ALT**	41.5±22.4	45.9±27.8	52.0±32.1	0.0946
**AST/ALT ratio**	0.73±0.20	0.91±0.32	1.51±1.25	<0.01
**GGT**	57.8±53.2	65.6±61.5	172.6±219.4	<0.01
**Albumin**	4.4±0.2	4.4±0.2	4.3±0.3	0.1697
**Total cholesterol**	213.8±35.0	208.4±34.4	187.8±32.5	<0.01
**Triglyceride**	150.6±86.5	152.5±99.0	172.7±136.4	0.9267
**HDL-C**	53.2±10.8	54.8±11.9	57.7±15.0	0.1211
**Glucose**	122.9±35.5	120.7±31.9	121.6±32.9	0.5514
**Creatinine**	0.82±0.42	0.84±0.22	0.88±0.23	0.0824
**Uric acid**	6.0±1.3	6.0±1.3	5.7±1.2	0.364
**Iron**	111.5±36.5	116.3±37.8	147.5±63.3	<0.01
**Platelet count**	246.2±47.8	190.0±35.8	144.8±35.7	<0.01
**FIB-4 index**	0.95±0.21	1.69±0.34	3.92±1.61	<0.01
**Fuc-Hpt**	168.3±194.0	200.2±231.7	447.7±556.5	<0.01

Data are presented as the mean ± SD.

Abbreviations: SBP, systolic blood pressure; DBP, diastolic blood pressure; HDL-C, high density lipoprotein cholesterol.

* *P* values correspond to the comparison among groups. Kruskal-Wallis test for continuous factors and Pearson's Chi-square test for categorical factors were used.

## Discussion

Distinguishing NASH from non-NASH patients and monitoring NASH disease progression are extremely important in the clinical management of NAFLD. Above all, a noninvasive and reliable approach is needed in the field. In the present report, we show that serum Fuc-Hpt levels were significantly elevated in NASH patients compared with non-NASH patients. Ballooning hepatocytes are known as a typical pathological character of steatohepatitis including NASH [22], [23], [30]. The ability to detect the presence of ballooning hepatocytes is quite important in distinguishing NASH from simple steatosis. We show that serum Fuc-Hpt levels undergo a stepwise increase with increasing hepatocyte ballooning scores in biopsy-proven NAFLD patients in our study. In addition, Fuc-Hpt levels were significant and independent determinants for the discrimination of the presence of ballooning hepatocytes, even after adjustment for age, BMI, serum levels of AST, GGT, total cholesterol, triglyceride, IRI, ferritin, platelet count, and caspase-cleaved cytokeratin-18 (M30 antigen). Moreover, measurement of serum Fuc-Hpt concentrations was superior compared to measurement of the M30 antigen in distinguishing NASH patients from non-NASH patients and predicting both the presence of ballooning hepatocytes and fibrosis severity in NAFLD patients. Serum Fuc-Hpt levels can serve as a novel diagnostic biomarker for NASH.

Very recently, we reported that serum Mac-2 binding protein (Mac-2bp) levels constitute a superior biomarker for distinguishing NASH from non-NASH patients (manuscript in press). Mac-2bp is a glycoprotein that has seven potential *N*-glycosylation sites [35], [36], and the *N*-glycans in Mac-2bp are susceptible to fucosylation [37]. We also found that Mac-2bp levels had a positive correlation with hepatocyte ballooning scores and significantly increased with increasing hepatocyte ballooning scores. We have reported previously that many glycoproteins in bile were strongly fucosylated compared to serum glycoproteins, and suggested that fucosylation may be a possible signal for the polarized secretion of glycoproteins into bile in the liver [20].

Indeed, we recently showed that fucosylated alpha-fetoprotein is more selectively secreted into bile [38]. Therefore, we hypothesized the reason for the elevation of serum Fuc-Hpt levels in NASH patients as follows. An increase in ballooning hepatocytes, which lose polarized secretion of fucosylated glycoproteins, would induce disruption of the fucosylation-based machinery. As a consequence, the secretion of Fuc-Hpt into the serum would increase, and serum Fuc-Hpt levels would become elevated in NASH patients. On the other hand, M30, cytokeratin-18 fragment, is produced from apoptotic hepatocytes, and correlate with the magnitude of hepatocyte apoptosis [33]. The secretion mechanisms into sera are thought to be different between Fuc-Hpt and M30. We think that this is the reason why these two serum proteins did not correlate each other. In the present study, we found that serum Fuc-Hpt levels were increased in NASH patients and that Fuc-Hpt levels were elevated with increasing hepatocyte ballooning scores. In addition, ballooning hepatocytes are also a typical pathological characteristic in alcoholic liver diseases, and Chambers et al. previously reported that serum Fuc-Hpt levels assessed by Western blotting were elevated in patients with alcoholic liver diseases [25]. These results would enhance the significance of our hypothesis regarding the elevation of fucosylated glycoproteins in NASH patients. Haptoglobin is a glycoprotein produced mainly in the liver, and is abundant in serum. The use of serum Fuc-Hpt levels to predict hepatocyte ballooning scores in NAFLD patients is a novel type of biomarker in NASH diagnosis. As far as we know, no serum biomarkers can predict hepatocyte ballooning scores with sufficient accuracy comparable to Fuc-Hpt.

In our study, there were a number of patients with low serum Fuc-Hpt levels even in the patients with high hepatocyte ballooning score. Therefore, the complete selection of cutoff value as a biomarker of NASH, hepatocyte ballooning score, and liver fibrosis degree is somewhat difficult at this time. We think there were at least two mechanisms that would explain the wide range of serum Fuc-Hpt levels in the patients with high hepatocyte ballooning score. First, haptoglobin has four potential *N*-glycosylation sites [17], and it has been reported that an *N*-glycan at a specific site plays a pivotal role in apical sorting in a glycoprotein possessing multiple *N*-glycans [39]. Site-specific fucosylation in the haptoglobin *N*-glycan might correlate with the elevation of serum Fuc-Hpt levels in NASH patients. Site-directed oligosaccharide analysis with mass spectrometry should be conducted to elucidate the features of the fucosylation site in NASH patients. Second, normal hepatocytes do not express fucosylation regulatory genes, while their expression is increased in hepatocellular carcinoma cells [38]. Recently, it is reported that fucosylation on serum alpha 1-acid glycoprotein is associated with liver fibrosis, suggesting that non-cancerous hepatocytes with high levels of fucosylation regulatory genes might lead to the secretion of fucosylated proteins into serum [40]. The hepatocytes of the patients with low serum Fuc-Hpt levels and frequent ballooning hepatocyte would have low fucosylation regulatory genes, and produce small amount of Fuc-Hpt in their hepatocytes. To elucidate this issue, further investigations should be required in our future study. In our present study, we adopted several cutoff values to examine the availability of Fuc-Hpt. We found that setting cutoff value to the mean of normal control plus 1 SD (484.3 U/mL) was able to distinguish NASH patients from simple steatosis patients with sufficient specificity and that this cutoff value increased the number of false negative subjects. However, at this time, we think that the best cutoff value is 484.3 U/mL to rule out false positive patients for distinguishing NASH patients from simple steatosis patients.

This study has several limitations. First, the proportion of non-NASH patients (14.5%) was small compared with that of NASH patients (85.5%) in the biopsy-proven NAFLD patients study. Second, patients were recruited from hepatology centers in Japan with a particular interest in studying NAFLD, and the possibility of some referral bias could therefore not be ruled out. A patient selection bias might also have existed, because liver biopsy might have been considered for NAFLD patients who were likely to have NASH. The findings of the biopsy-proven NAFLD study may thus not represent NAFLD patients in the general population. However, the increase in serum Fuc-Hpt levels with the FIB-4 index in the health checkup study would enhance the significance of serum Fuc-Hpt level measurement in the general population. Third, there were 15 differences among 126 liver specimens in scoring for hepatocyte ballooning between two hepatic pathologists at first in our present study. Interobserver variation in defining ballooning hepatocytes is one of the most important concerning in the NASH diagnosis [31], [41]–[43]. Recently, Lackner et al. proposed more stringent definition of hepatocyte ballooning using keratin 8/18 immunohistochemistry, and loss of keratin 8/18 immunostaining can serve as an objective marker of a specific type of ballooning hepatocytes [32], [44]. In this study, we diagnosed hepatocyte ballooning score using HE stained liver specimens. Therefore, the diagnosis of these samples was carefully discussed and made by consensus between two hepatic pathologists (YK and HF).

We conclude, despite these limitations, that serum Fuc-Hpt levels can distinguish NASH from non-NASH patients and estimate the increase in hepatocyte ballooning scores of NAFLD patients with an accuracy superior to that of the M30 antigen in our patients. Further investigation is needed using a larger number of biopsy-proven NAFLD patients.

## Supporting Information

Figure S1 (TIF)

Table S1 **Distribution of parameters according to Matteoni’s classification in the biopsy-proven NAFLD patients.**
(DOCX)

Table S2 **Histological Characteristics of the biopsy-proven NAFLD patients.**
(DOCX)

Table S3 **Correlation coefficients of relationships between serum Fuc-Hpt levels and various parameters in the biopsy-proven NAFLD patients.**
(DOCX)

Table S4 **Various Fuc-Hpt cutoff values for the detection of NASH, the presence of ballooning hepatocyte, and advanced fibrosis.**
(DOCX)

Table S5 **Multiple logistic regression analysis of factors associated with F3 (diagnosed by FIB-4 index).**
(DOCX)
