# Supplementary figures and images for: Serum Fucosylated Haptoglobin as a Novel Diagnostic Biomarker for Predicting Hepatocyte Ballooning and Nonalcoholic Steatohepatitis
Source: PLoS One. 2013 Jun 21;8(6):e66328. doi: 10.1371/journal.pone.0066328 (PMC3689816; doi:10.1371/journal.pone.0066328)

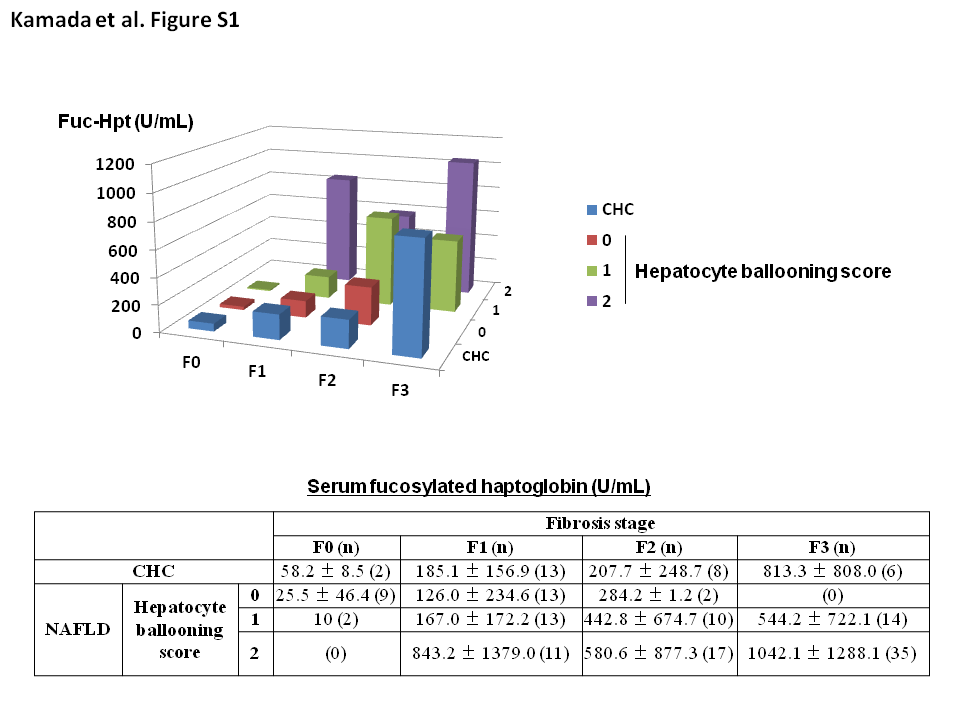

Supplement: Figure S1 — (TIF) [file pone.0066328.s001.tif]
