# Supplementary material for: Serum Fucosylated Haptoglobin as a Novel Diagnostic Biomarker for Predicting Hepatocyte Ballooning and Nonalcoholic Steatohepatitis
Source: PLoS One. 2013 Jun 21;8(6):e66328. doi: 10.1371/journal.pone.0066328 (PMC3689816; doi:10.1371/journal.pone.0066328)
Supplement: Table S1 — Distribution of parameters according to Matteoni’s classification in the biopsy-proven NAFLD patients. (DOCX) [file pone.0066328.s002.docx]

**Table S1. Distribution of parameters according to Matteoni’s classification in the biopsy-proven NAFLD patients**

| **Matteoni's classification** | **Type 1** | **Type 2** | **Type 3** | **Type 4** |
| --- | --- | --- | --- | --- |
| **number** | 8 | 11 | 15 | 92 |
| **Gender (M/F)** | 6/2 | 7/4 | 9/6 | 48/44 |
| **BMI (kg/m^2^)** | 27.8 ± 5.4 | 26.3 ± 2.9 | 27.2 ± 4.6 | 27.6 ± 5.3 |
| **ALT (U/L)** | 67.9 ± 48.3 | 74.8 ± 51.3 | 112.7 ± 95.4 | 98.0 ± 71.2 |
| **IRI (mU/mL)** | 9.9 ± 6.2 | 8.9 ± 4.0 | 13.9 ± 14.1 | 14.4 ± 11.5 |
| **Hyaluronic acid (ng/dL)** | 28.2 ± 21.3 | 25.2 ± 15.6 | 28.1 ± 23.7 | 90.0 ± 101.9 |
| **Fuc-Hpt (U/mL)** | 193.5 ± 284.1 | 51.2 ± 87.4 | 469.9 ± 1052.3 | 686.3 ± 1021.5 |
| **M30 antigen (U/L)** | 401.0 ± 407.9 | 699.1 ± 559.1 | 426.9 ± 246.2 | 925.6 ± 854.3 |

Data are presented as the mean ± SD.

Abbreviations: See Table 1.
