# Supplementary material for: Serum Fucosylated Haptoglobin as a Novel Diagnostic Biomarker for Predicting Hepatocyte Ballooning and Nonalcoholic Steatohepatitis
Source: PLoS One. 2013 Jun 21;8(6):e66328. doi: 10.1371/journal.pone.0066328 (PMC3689816; doi:10.1371/journal.pone.0066328)
Supplement: Table S2 — Histological Characteristics of the biopsy-proven NAFLD patients. (DOCX) [file pone.0066328.s003.docx]

**Table S2. Histological Characteristics of the biopsy-proven NAFLD patients**

| **Factor** | **Subjects number (%)** |
| --- | --- |
| **All subjects (n)** | 126 |
| **Steatosis** |  |
| **5-33%** | 59 (46.8) |
| **33-65%** | 46 (36.5) |
| **>65%** | 21 (16.7) |
| **Lobular Inflammation** |  |
| **None** | 2 (1.6) |
| **<2 under 200x** | 69 (54.8) |
| **2-4 under 200x** | 42 (33.3) |
| **>4 under 200x** | 13 (10.5) |
| **Ballooning hepatocytes** |  |
| **None** | 24 (19.0) |
| **Few** | 39 (31.0) |
| **Many** | 63 (50.0) |
| **Fibrosis** |  |
| **0** | 11 (8.9) |
| **1** | 37 (29.8) |
| **2** | 28 (22.6) |
| **3&4** | 48 (38.7) |
| **NAFLD activity score (NAS)** |  |
| **0** | 0 (0) |
| **1** | 2 (1.6) |
| **2** | 9 (7.1) |
| **3** | 19 (15.1) |
| **4** | 25 (19.8) |
| **5** | 41 (32.5) |
| **6** | 22 (17.5) |
| **7** | 8 (6.3) |
| **8** | 0 |
