# Supplementary material for: Serum Fucosylated Haptoglobin as a Novel Diagnostic Biomarker for Predicting Hepatocyte Ballooning and Nonalcoholic Steatohepatitis
Source: PLoS One. 2013 Jun 21;8(6):e66328. doi: 10.1371/journal.pone.0066328 (PMC3689816; doi:10.1371/journal.pone.0066328)
Supplement: Table S3 — Correlation coefficients of relationships between serum Fuc-Hpt levels and various parameters in the biopsy-proven NAFLD patients. (DOCX) [file pone.0066328.s004.docx]

**Table S3. Correlation coefficients of relationships between serum Fuc-Hpt levels and various parameters in the biopsy-proven NAFLD patients**

| **Factor** | ***r*** | ***P* value** |
| --- | --- | --- |
| **Age** | 0.14 | <0.05 |
| **BMI** | -0.024 | 0.79 |
| **AST** | 0.15 | 0.086 |
| **ALT** | -0.052 | 0.56 |
| **AST/ALT ratio** | 0.31 | <0.01 |
| **GGT** | 0.13 | 0.16 |
| **Total cholesterol** | -0.24 | <0.01 |
| **Triglyceride** | -0.15 | 0.11 |
| **Glucose** | 0.067 | 0.46 |
| **IRI** | -0.057 | 0.56 |
| **Albumin** | -0.25 | <0.01 |
| **Ferritin** | 0.018 | 0.051 |
| **Platelet count** | -0.32 | <0.01 |
| **Hyaluronic acid** | 0.55 | <0.01 |
| **FIB-4 index** | 0.40 | <0.01 |
| **M30 antigen** | 0.056 | 0.54 |
| **Haptoglobin** | -0.27 | <0.01 |
| **NAS total** | 0.28 | <0.01 |
| **Steatosis** | -0.16 | 0.075 |
| **Inflammation** | 0.26 | <0.01 |
| **Ballooning** | 0.41 | <0.01 |
| **Fibrosis** | 0.41 | <0.01 |
