# Supplementary material for: Serum Fucosylated Haptoglobin as a Novel Diagnostic Biomarker for Predicting Hepatocyte Ballooning and Nonalcoholic Steatohepatitis
Source: PLoS One. 2013 Jun 21;8(6):e66328. doi: 10.1371/journal.pone.0066328 (PMC3689816; doi:10.1371/journal.pone.0066328)
Supplement: Table S4 — Various Fuc-Hpt cutoff values for the detection of NASH, the presence of ballooning hepatocyte, and advanced fibrosis. (DOCX) [file pone.0066328.s005.docx]

**Table S4. Various Fuc-Hpt cutoff values for the detection of NASH, the presence of ballooning hepatocyte, and advanced fibrosis.**

(A) Fuc-Hpt cutoff values for NASH prediction.

| **Method for cutoff value** | **Cutoff value (U/mL)** | **Sensitivity (%)** | **Specificity (%)** | **Accuracy (%)** |
| --- | --- | --- | --- | --- |
| **Youden index (ROC curve)** | 36.1 | 69.16 | 73.68 | 69.84 |
| **Mean* + 1 SD** | 378.8 | 39.25 | 89.50 | 46.83 |
| **Mean +2 SD** | 589.8 | 31.80 | 89.50 | 40.48 |
| **Upper limit of normal control** | 780.9 | 23.36 | 100 | 34.92 |
| **Upper limit of simple steatosis** | 677 | 27.1 | 100 | 38.1 |

*Mean value of normal control subjects.

(B) Fuc-Hpt cutoff values for ballooning hepatocyte prediction.

| **Method for cutoff value** | **Cutoff value (U/mL)** | **Sensitivity (%)** | **Specificity (%)** | **Accuracy (%)** |
| --- | --- | --- | --- | --- |
| **Youden index (ROC curve)** | 36.1 | 71.57 | 75.00 | 72.20 |
| **Mean + 1 SD** | 378.8 | 41.18 | 91.67 | 50.79 |
| **Mean +2 SD** | 589.8 | 33.33 | 91.67 | 44.44 |
| **Upper limit of normal control** | 780.9 | 24.51 | 100 | 38.89 |
| **Upper limit of simple steatosis** | 677 | 28.43 | 95.83 | 41.27 |

(C) Fuc-Hpt cutoff values for advanced fibrosis prediction.

| **Method for cutoff value** | **Cutoff value (U/mL)** | **Sensitivity (%)** | **Specificity (%)** | **Accuracy (%)** |
| --- | --- | --- | --- | --- |
| **Youden index (ROC curve)** | 38.8 | 76.92 | 62.50 | 71.43 |
| **Mean + 1 SD** | 378.8 | 44.87 | 81.25 | 58.73 |
| **Mean +2 SD** | 589.8 | 38.46 | 87.50 | 57.14 |
| **Upper limit of normal control** | 780.9 | 29.49 | 95.83 | 54.76 |
| **Upper limit of simple steatosis** | 677 | 33.33 | 91.67 | 55.56 |
