# Supplementary material for: Serum Fucosylated Haptoglobin as a Novel Diagnostic Biomarker for Predicting Hepatocyte Ballooning and Nonalcoholic Steatohepatitis
Source: PLoS One. 2013 Jun 21;8(6):e66328. doi: 10.1371/journal.pone.0066328 (PMC3689816; doi:10.1371/journal.pone.0066328)
Supplement: Table S5 — Multiple logistic regression analysis of factors associated with F3 (diagnosed by FIB-4 index). (DOCX) [file pone.0066328.s006.docx]

**Table S5. Multiple logistic regression analysis of factors associated with F3 (diagnosed by FIB-4 index)**

|  |  | **95% CI** | |  |
| --- | --- | --- | --- | --- |
| **Factor** | **Odds ratio** | **Lower** | **Upper** | ***P* value** |
| **BMI** | 0.951 | 0.852 | 1.05 | 0.3361 |
| **SBP** | 1.379 | 0.606 | 2.973 | 0.4307 |
| **Total cholesterol** | 0.9833 | 0.972 | 0.995 | <0.05 |
| **Triglyceride** | 1.002 | 0.998 | 1.005 | 0.2919 |
| **Creatinine** | 1.361 | 0.371 | 2.28 | 0.4883 |
| **iron** | 1.015 | 1.007 | 1.023 | <0.01 |
| **Fuc-Hpt** | 1.187 | 1.074 | 1.313 | <0.01 |

**Supporting Information Legends**

**Figure S1**

Upper figure represented the mean serum Fuc-Hpt levels classified with the hepatocyte ballooning score and the fibrosis stage in NAFLD and CHC patients. Lower table represented the mean ± SD value of serum Fuc-Hpt levels. The numbers shown in parentheses are the number of patients applied into the categories classified with the hepatocyte ballooning score and fibrosis stage.
